# Mobile applications for ophthalmology: a quantitative and qualitative analysis

**DOI:** 10.1186/s12913-026-15199-2

**Published:** 2026-07-23

**Authors:** Shahla Faramarzi, Kosar Ghaddaripouri, Parmida Sanaei, Zahra Zare, Azita Yazdani

**Affiliations:** 1 Department of Health Information Management, School of Health Management and Information Sciences, Iran University of Medical Sciences, Tehran, Iran; 2 Student Research Committee, Shiraz University of Medical Sciences, Shiraz, Iran; 3 Present Address: Health Human Resources Research Center, School of Health Management and Information Sciences, Shiraz University of Medical Sciences, Shiraz, Iran; 4 Present Address: Clinical Education Research Center, Shiraz University of Medical Sciences, Shiraz, Iran; 5 Present Address: Department of Medical Informatics and Health Information Technology, School of Health Management and Information Sciences, Shiraz University of Medical Sciences, Shiraz, Iran

**Keywords:** MARS, Application, Mobile health, Vision, Eye, m-health, Ophthalmology

## Abstract

**Objective:**

This study aimed to evaluate the quality of ophthalmology-related mobile health applications (mHealth apps) available in Persian and English across Android and iOS platforms.

**Method:**

A structured review of apps related to ophthalmology was conducted in four mobile app stores (Cafe Bazaar, Google Play, App Store, Myket) from July 31 to August 15, 2024, using relevant English and Persian keywords. The quality of selected apps was independently assessed by three reviewers using the Mobile App Rating Scale (MARS). Clinical evidence was also searched in PubMed, Scopus, and Google Scholar.

**Result:**

Out of 4,968 ophthalmology-related apps initially identified, 37 apps met the inclusion criteria for final quality assessment—4 in Persian and 33 in English. According to the MARS scale, the Functionality domain had the highest average score (4.24), followed by Aesthetics (3.70), Engagement (3.22), and Information (3.13). Clinical effectiveness evidence was found for the “WHO eyes” app. The Persian app “چشم یار” achieved the highest average score of 4.42, and the English app “Eye Patient” received the highest average score of 4.83.

**Conclusion:**

This study demonstrates that the quality of ophthalmology-related apps meets acceptable criteria according to the MARS scale; however, user engagement and the quality of the information provided remain suboptimal. These results highlight the need for improving interactive features, motivational design elements, and evidence-based educational content to enhance the overall effectiveness of ophthalmology-related mHealth apps.

**Supplementary Information:**

The online version contains supplementary material available at https://doi.org/10.1186/s12913-026-15199-2.

## Introduction

Visual impairment is a major global health challenge with substantial social and economic consequences. It affects low-resource countries through limited access to care for preventable causes and high-income regions via rising age-related conditions like cataracts and glaucoma. This growing demand highlights the need for accessible self-monitoring tools to enable early detection and timely intervention [1, 2]. With the rapid progress in digital technology and the increasing penetration of smartphones, ophthalmology has successfully integrated mobile apps for self-monitoring of visual functions at home. Numerous smartphone apps have been developed to aid in the diagnosis, treatment, and symptom management of ophthalmic conditions [3]. Ophthalmology is increasingly integrating telehealth into its practice, and the application of mHealth tools holds significant potential to transform clinical workflows in this field [4–6].

Recent studies have highlighted concerns about the quality, reliability, and clinical applicability of mobile apps designed for visual acuity testing. Evaluations have shown that many such apps lack sufficient reliability for telehealth purposes, while only a limited number meet the standards required for routine clinical practice [7]. These results point to ongoing limitations in the accuracy, consistency, and overall performance of available ophthalmology mobile apps.

However, despite the increasing availability of mobile apps for ophthalmology, evaluations of their overall quality, functionality, and clinical relevance remain limited, particularly for Persian- or Iranian-developed apps, which have not yet undergone such assessments. The MARS is a validated, multidimensional tool developed to provide a reliable and objective way to assess the quality of mHealth apps. It is the most frequently used scale to rate health apps in research [8]. This structured review aims to apply the MARS to comprehensively assess the quality of currently available ophthalmology-related mobile apps in Persian and English. Persian-language ophthalmology apps deserve special attention because language is a major determinant of usability, accessibility, and health literacy in digital health interventions [9]. Globally, at least 2.2 billion people are affected by near or distance vision impairment [10]. According to a meta-analysis by Afarid et al. (2020), the pooled prevalence of visual impairment in Iran is 5.57% [11]. Although English-language apps are widely available and abundant globally, many Persian-speaking users encounter substantial barriers to benefiting from them, stemming from challenges in health literacy (particularly when using a non-native language), inadequate cultural adaptation, and limitations in language proficiency—especially in comprehending specialized ophthalmic terminology and instructions [12, 13]. This linguistic and cultural mismatch highlights the pressing need to evaluate the availability, quality, and cultural appropriateness of Persian-language ophthalmology apps to promote equitable access to vision care tools. Furthermore, to our knowledge, no prior study has systematically assessed Persian-language ophthalmology apps, representing an important evidence gap.

The present investigation described the principal attributes, core functions, and professional underpinnings of ophthalmology-related mobile apps, while simultaneously assessing their overall quality. Therefore, this study can provide valuable guidance for eye care professionals, designers, researchers, and other users in selecting evidence-based and effective apps for vision education and care.

## Methods

A structured app-stores search was conducted according to the Preferred Reporting Items for Systematic Reviews and Meta-Analyses (PRISMA) checklist [14]. Although this was not a formal systematic review, our methodological approach was informed by PRISMA guidelines, and relevant items from the checklist were followed to enhance the transparency and quality of reporting.

Due to the unique nature of mHealth app store databases, which differ substantially from traditional academic literature databases, certain deviations from the PRISMA guidelines were inevitable. Notably, app store search algorithms lack transparency, and the results displayed can change over time, which significantly limits the reproducibility of the search process [15].

This review was prospectively registered in the International Prospective Register of Systematic Reviews (PROSPERO) under registration number CRD420261412468. The registration record is available at:

https://www.crd.york.ac.uk/PROSPERO/view/CRD420261412468.

### Research app stores

A structured search was conducted in the App Store, Google Play, Cafe Bazaar, and MyKet app stores. The latter two are Iranian app stores. The Apple App Store serves as a safe and reliable platform that provides access to millions of applications with strong emphasis on privacy and security [16]. Google Play is an online digital distribution platform operated by Google, allowing users to download and install various digital content, including apps, games, movies, books, and music on their devices. Available on the Android operating system, the platform can be accessed via smartphones, tablets, and PCs [17]. Cafe Bazaar, the leading Persian-language app marketplace, is recognized as the most comprehensive repository of Android games and apps in Iran [18]. Iranian Android app stores, Cafe Bazaar and MyKet, demonstrate significant market penetration. According to 2022 data, Cafe Bazaar has 1.8 billion installs across hundreds of thousands of apps and games, with 45 million active installs and over 14 million purchasing users. MyKet, another prominent Android marketplace in Iran, reported that by 2024 it would have over 50 million active installs and 26 million monthly active users. Currently, the platform hosts approximately 350,000 Android apps and games, developed by over 22,000 developers [19].

### Selection of mobile apps

The initial search and review of apps were conducted from July 31 to August 15, 2024. All app store searches were conducted using devices physically located in Iran with locally registered Iranian Apple IDs and Google Play accounts. This approach was adopted due to the geographic location of the research team and associated practical constraints.

In this study, mobile apps were searched using keywords in both English and Persian. Table 1 presents the Persian keywords and their English equivalents used in the apps search strategy.

**Table 1:** Keywords included in the search

Persian keywords:	English keywords:
چشم	Eye, Vision, Ocular
چشم پزشکی	Ophthalmology

Each keyword was entered individually into the search bar of each app store. The evaluators read the descriptions of the app stores and manually reviewed them against the inclusion and exclusion criteria. Additionally, one team member (SF) manually collected app store ratings and the number of user ratings from the app stores.

#### Inclusion criteria

Apps were included if they met the following criteria:

- The language of the apps must be Persian or English.
- The app should be free and able to be installed and run on a smart mobile phone.

#### Exclusion criteria

Apps were excluded if they met any of the following criteria:

- Apps that required in-app purchases to access additional features.
- Games were excluded from the study.
- Apps that were not publicly available, or in other words, were only available to a specific group of users and other users could not download and use them, and required a special identification to log in.
- Completely static apps (such as PDF viewers) were removed.
- Apps that were solely related to adjusting the brightness of the phone and had no other functionality.
- Apps that were related to selling glasses.
- The apps that were inactive.

### Analysis method

After removing the duplicates, the retrieved apps were screened based on the title and description. The apps retrieved from the previous step were then installed on the raters’ phones. Based on the inclusion and exclusion criteria and after a thorough review of the content and features, the selected apps entered the final stage of quality evaluation.

The MARS, a widely utilized tool for evaluating the quality and content of mHealth apps, was employed to assess the quality of the apps. MARS is a multidimensional instrument designed to appraise the overall merit of mHealth apps [20]. Its user-friendly format allows the raters to evaluate Engagement, Functionality, Aesthetics, and Information quality effectively [21]. The scale comprises 23 items divided into three sections. The objective section, which measures overall quality, includes 19 items categorized into four domains: Engagement, Functionality, Aesthetics, and Information quality (sections A to D) [22]. The mean scores of these domains constitute the overall quality score of the app. Subjective quality is assessed via section E, consisting of four questions that evaluate user satisfaction. Section F measures the app’s impact on users, such as behavioral change. Ratings were assigned on a 5-point Likert scale, where 1 indicates inadequate, and 5 indicates excellent performance. Multiple independent raters evaluated each app across predefined dimensions, with ratings recorded in an Excel sheet and averaged per dimension. The overall quality score was computed by averaging the scores across sections A to D, while Subjective quality and Perceived impact were obtained by averaging sections E and F, respectively. A final mean score was calculated to represent the overall quality and performance of each app within this study.

To examine clinical evidence related to the effectiveness of the final apps, we searched the names of these apps without time limits in PubMed, Scopus, and Google Scholar to identify all studies conducted on these apps. For each app, the exact name of the app was searched using the following strategy: [Exact app name] AND (“app” OR “apps” OR “application” OR “mobile application” OR “smartphone application” OR “mHealth” OR “mobile health”).

### The MARS raters

The quality of the selected apps was independently evaluated by three expert raters (KG, PS, ZZ), all of whom were specialists in medical informatics and health information technology with more than one year of experience in health technology assessment. They were proficient in using the MARS and familiar with the relevant ophthalmology literature. Disagreements between the reviewers were resolved through structured discussion and consensus meetings. In the few instances where consensus was not immediately reached on specific items, the supervising evaluator (AY) was consulted to finalize the rating. The senior evaluator had 15 years of experience in user interface design and 10 years of professional activity in medical informatics. This consensus-based approach was applied rigorously to ensure consistency and minimize subjectivity. The raters assessed each app individually. Intra-class correlation (ICC) was used to estimate IRR for the MARS tool. The ICC coefficient was calculated with a two-way random model and for agreement level for all apps.

## Result

### Systematic search results

**Fig. 1:**
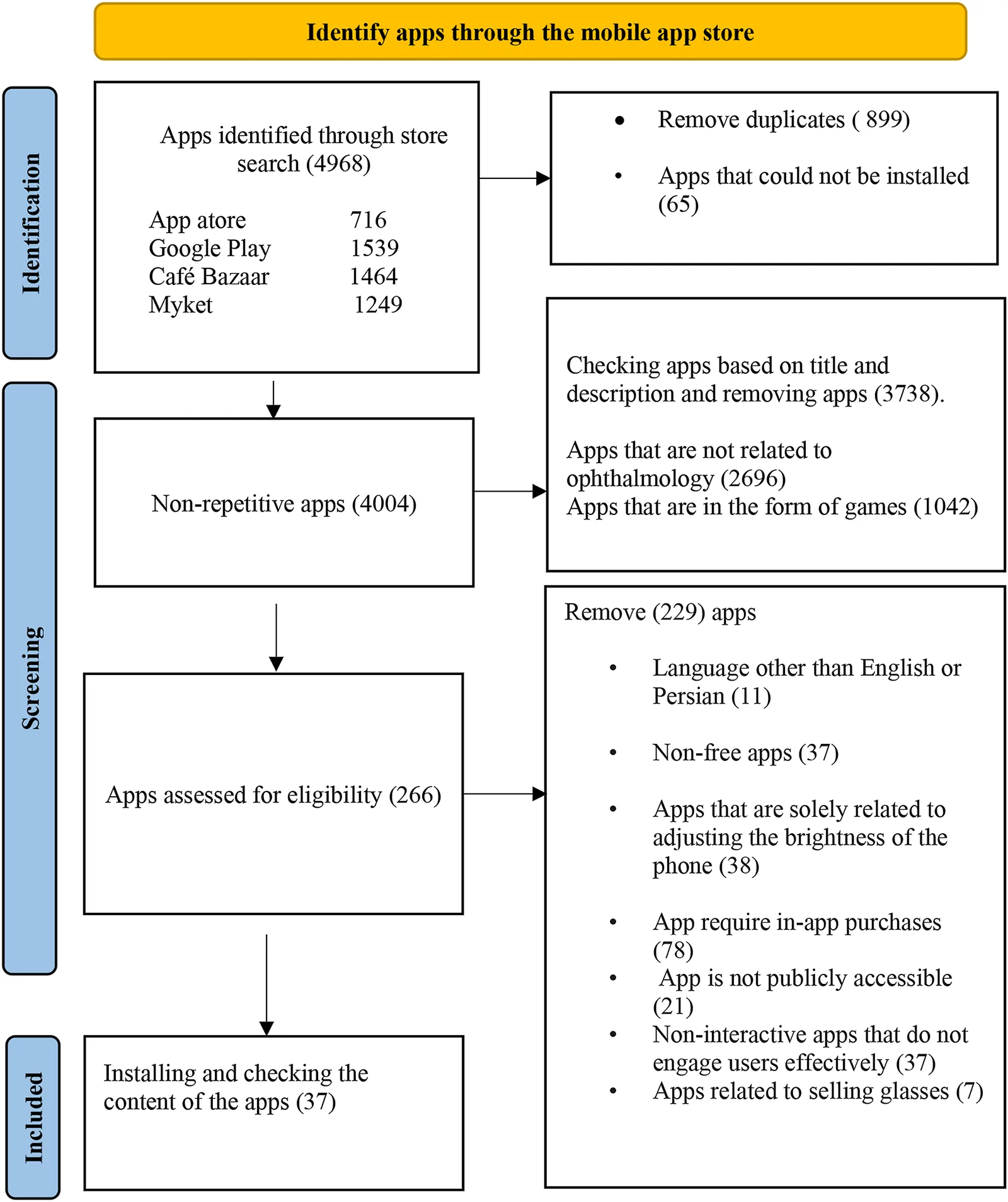
Steps to choose companion apps from different stores

As shown in Fig. 1, a total of 4,968 apps were found. 1895 apps with the Persian keyword and 3,073 apps with the English keywords were extracted from the App Store, Google play, Cafe Bazaar and Myket stores. In the first screening stage, after consolidating the results, 899 duplicate apps were removed, and 65 apps were excluded from the study because they failed to open on four Android devices. By reviewing the titles and descriptions, 2,696 apps unrelated to ophthalmology and 1,042 apps in the form of games were excluded, leaving 266 apps to proceed to the next stage. The evaluators thoroughly assessed the remaining 266 apps based on payment model, availability, engagement, and language. In the end, 37 apps entered the final stage of the study.

The selection process for mobile apps, stratified by app store, is detailed in four PRISMA flow diagrams in the Supplementary Files (Supplementary 1–4).

### MARS scale

Table 2 presents the evaluation results based on the MARS scale. In general, the MARS domain scores indicate higher performance in Functionality compared with other domains. Overall, the results suggest that the included applications perform well in terms of technical functionality, yet they are comparatively weaker in sustaining user engagement and providing high-quality, evidence-informed information.

**Table 2:** Evaluation results based on the MARS scale

#	App name	Engagement Mean Score (A)	Functionality Mean Score (B)	Aesthetics Mean Score (C)	Information Mean Score (D)	App subjective quality Score (E)	App perceived impact Score (F)	App quality mean Score
**1**	Optocharts _ all eye test	4	2.75	3	3.57	2	3	3.33
2	Eye exam	2.4	4	1.33	1.85	1.5	2	2.39
3	تست بینایی	3.6	5	3.66	3.28	2.75	2	3.88
4	Eye exercises & eye trainer	2.6	4.5	4.3	3	2.75	3.16	3.6
5	Eye care pro – eye exercises 20 20 20, eye filter	3.2	5	5	3.57	3.5	4	4.19
6	eye exercises	2.8	4.5	4.3	3.28	4	4.16	3.72
7	eye exercise - eye workout & eye training plans	3.8	5	5	3.42	4	3.5	4.30
8	eye rest -Reminder : eye exercises and breaks	2.6	3.5	3	3.28	2.75	3.16	3.09
9	REST rapid eye screening test	2.4	4.25	3.3	2.42	2	3.5	3.09
10	eye exercises : workout vision	3.4	4.75	4.60	3.14	3.5	3.16	3.97
11	vision training & eye exercise	3.4	3.5	4	2.14	2.5	2.66	3.26
12	BLINK : eye blinking -Reminder	3	3.75	3.33	2.85	2	2.66	3.23
**13**	Eye protection	2.2	3.75	3	2.85	2	2.16	2.90
14	eye doctor. Eye stra. 20-20-20	2.4	4	3.33	2.85	2.75	2.66	3.14
15	eye care : eye, test, exercise & bluelight filter	3.2	3.75	4	3.42	3.5	3.33	3.59
16	بهبود بینایی : تمرین چشم ، تست بینایی	3.8	4.5	4	3	3.25	3.33	3.82
17	Vision tests	3.4	4.75	4	2.71	4.25	4.33	3.71
18	Tumblig E chart	2.4	3.25	3.33	2.85	2.25	3	2.95
19	چشم یار	2.8	4.25	3.66	3	2.30	2.66	4.42
20	دکتر پلاس ( بیماری های چشم )	3	4.25	2.33	2.57	3.75	4	3.03
21	Who eyes	2.60	4.50	3.66	3.57	2	2.33	3.58
22	Eye health manager	3.4	4	2.33	3.71	3.50	3.16	3.36
**23**	Vision to vision	2.60	4.25	4.33	4.42	2	3	3.96
24	Eye patient	5	4.5	5	4.85	4.25	4.66	**4.83**
25	Pristine eye hospitals	4.8	3.5	5	3.42	2.25	2.66	4.18
26	AR Eye	3.2	4.25	4	2.71	4	3.66	3.54
27	Uveitisoncall	3.6	5	3.33	3	3.75	4.16	3.73
28	Ocularcheck: acuity exam	4	5	4	3.71	4	3	4.17
29	Glaucoma simulator	2.60	3.75	3.33	2.71	2.25	3.16	3.09
30	SUPRACOR™ Advisor	3.4	4.75	4	3.71	3.75	3.66	3.96
31	Cataract lens	3	5	4	3.57	3.25	3.66	3.89
32	Visionstation	2	2	2.33	2.28	1.25	1	2.15
33	Visioncare- eye exam	4.2	4.75	4.33	3	4.25	3.5	4.07
34	Glaucoma notebook – IOP Note	3.2	5	2.66	3.14	4.5	3	3.50
35	Complexcataract	3.60	5	4	2.71	3.75	3.5	3.81
36	eyeRx	3.66	5	4.66	3	5	4	4.06
37	Eyedea refractive	4	3.5	3.33	3.14	4.75	4.16	3.49
Total average		3.22	**4.24**	3.70	3.13	3.13	3.21	3.59

The “Eye Patient” app received the highest average score of five in the Engagement domain, while the “Visionstation” app received the lowest average score. In the Functionality domain, the review showed that the “تست بینایی”, “Eye care pro – eye exercises 20 20 20, eye filter”, “Eye Exercise - Eye Workout & Eye Training Plans”, “UveitisOnCall”, “OcularCheck: Acuity Exam”, “Cataract Lens”, “Glaucoma Notebook – IOP Note”, “ComplexCataract”, and “eyeRx” apps, all received the highest average score of five, whereas “Visionstation” app received the lowest average score of two.

In the Aesthetics domain, the “Eye Care Pro – Eye Exercises 20 20 20, “Eye Filter”, “Eye Exercise - Eye Workout & Eye Training Plans”, “Eye Exercises _ Workout Vision”, “Eye Patient”, and “Pristine Eye Hospitals” apps all achieved the highest average score of five, while the app “Eye Exam” received the lowest average score of 1.33.

In the Information domain, “Eye Patient” received the highest average score of 4.85, and “Eye Exam” received the lowest average score of 1.85. Also, in the Subjective Quality domain, the “eyeRx” received the highest average score of five, while “Visionstation” received the lowest average score of 1.25. “Eye Patient” achieved the highest average score of 4.66 in Perceived Impact, whereas “Visionstation” had the lowest average score of one in this domain. Overall, “Eye Patient” received the highest mean quality score of 4.83, and “Visionstation” received the lowest mean quality score of 2.15.

After reviewing the clinical evidence related to the effectiveness of the final apps, article [23] provided preliminary evidence of the efficiency and usability of the “WHOeyes” app in visual acuity screening among the student population, which can serve as preliminary scientific support for the use of this app in population studies. For the other reviewed apps, no valid scientific evidence was found to support their use, and the developers did not provide any evidence demonstrating the effectiveness or usefulness of their apps.

### Technical information

The descriptive and technical characteristics of the evaluated ophthalmology mobile apps are presented in Table 3.

**Table 3:** Descriptive and technical information of the included apps

#	App name	Developer	Version	Version rank/ Ratings	Last update	Operating system	App targets	Strategies	Affiliations	Age group	Technical aspects of app
1	Optocharts _ all eye test	Shadi AboelNaga	6.0	0	2023/06/13	Android	- Physical health	-Assessment -feedback -Information, Education	UN	G	- Requires login -Needs internet access to function
2	Eye exam	Andrei Brusentsov	2.7	UN	2024/05/24	Android	- Physical health -Behavior change	-Assessment -monitoring, tracking -feedback	UN	G	-Needs internet access to function -Reminder
3	تست بینایی	ویلیام اپ	2.33	UN	2024/02/24	Android	- Physical health -Entertainment	-Feedback	UN	G	-Needs internet access to function -Allows sharing -
4	Eye exercises & eye trainer	phone cooler & mobile cleaner - speed check	1.2	4.3	2024/11/23	Android	- Physical health	-Assessment -Information, Education -Monitoring, Tracking -Advice, Tips, Strategies, Skills training	UN	G	-
5	Eye care pro – eye exercises 20 20 20, eye filter	bhavay goyal	0.7	5	2022/06/01	Android	-Behavioral change -Physical health -	- Assessment -Feedback -Information, education -Goal setting -Advice, tips, strategies, skills training	UN	G	-Needs internet access to function -Allows sharing -Reminder
6	eye exercises	DCOPR	2.5.1	0	2023/08/19	Android	-Behavioral change -Goal Setting -Physical health	-Assessment -Information, Education - Goal setting - Advice, Tips, Strategies, Skills training	UN	G	- Reminder
7	eye exercise - eye workout & eye training plans	tools application developer	1.6	0	2022/06/04	Android	-Goal Setting -Physical health	-Information, Education -Monitoring, Tracking - Goal setting - Advice, Tips, Strategies, Skills training	UN	G	-Needs internet access to function -Allows sharing ، - Reminder for eye exercise
8	eye rest -Reminder : eye exercises and breaks	yesil science	2.0.4	0	2021/11/05	Android	-Physical health -Behavior change	-Information/Education -Monitoring, Tracking -Advice, Tips, Strategies, Skills training -Relaxation	UN	G	- Eye Rest Reminder
9	REST rapid eye screening test	epic egg studio	1.0.2	0	2023/11/24	Android	Physical health	-Assessment -Information, Education	UN	G	-
10	Eye exercises _ workout vision	DZMITRY	1.2	UN	2024/01/16	Android 6.0 and up	-Physical health -Behaviour change	Information, Education -Assessment -Feedback -Advice, Tips, Strategies, Skills Training -Monitoring, Tracking -Goal setting	UN	G	-Reminders for eye rest
11	vision training & eye exercise	optics trainer	1.75	0	2024/11/22	Android	-Physical health -Behavior Change	-Assessment -Feedback -Information, Education -Advice, Tips, Strategies, Skills Training	UN	G	-
12	BLINK : eye blinking -Reminder	app ranger community	8	0	2021/08/10	Android	-Physical health -Behavior Change	- Advice, Tips, Strategies, Skills Training	UN	G	- Blink reminderr
13	Eye protection	kapron-ap	5.4.7	0	2024/09/28	Android	-Physical health -Behavior Change	-Information, Education -Advice, Tips, Strategies, Skills training	UN	UN	- Reminder to rest the eyes
14	eye doctor. Eye stra. 20-20-20	apollie software	4.01	0	2023/08/26	Android	-Physical health -Behavior Change	-Monitoring, Tracking -Information, Education -Advice, Tips, Strategies, Skills training -Goal setting	UN	UN	-Needs internet access to function -Reminder
15	eye care : eye, test, exercise & bluelight filter	wellness labs	1.6	0	2021/11/11	Android	-Physical health	-Assessment -Information, Education -Monitoring, Tracking -Advice, Tips, Strategies, Skills Training	UN	G	- eye care exersice reminder
16	بهبود بینایی : تمرین چشم ، تست بینایی	mohselite	3.21	4.4	2022/01/12	Android	-Physical health	- Assessment -Information, Education -Advice, Tips, Strategies, Skills Training -Feedback	UN	G	- Needs internet access to function -Allows sharing -Reminders for training
17	vision tests	playzileiro	1	0	2024/09/02	Android	-Physical health -Behaviour change	-Assessment -Information, education -monitoring, tracking - Feedback	UN	G	-Allows sharing -Needs internet access to function
18	tumblig E chart	fonlow	2.7.2	2.3	2024/12/21	Android	Physical health	-Assessment -Feedback -Information, education -Monitoring, tracking strategies	UN	G	-Needs internet access to function
19	چشم یار	کوالامین	1.1	5	2021/05/10	Android	Physical health	-Assessment -Feedback -Information, Education -Monitoring, Tracking -Advice, Tips, Strategies, Skills training	UN	G	-Needs internet access to function - Reminders for eye rest
20	دکتر پلاس ( بیماری های چشم )	سعادت جابری	2.4	5	2017/12/12	Android	Physical health	-Information -Education -Assessment	UN	G	-
21	who eyes	WHO	2.3.2	UN	2024	android 5.1 and up	Physical health	Assessment	NGO	G	-
22	Eye health manager	Nikita tiart	2.3.0.18	UN	2024/10/17	android 7.0 and up	Physical health	Assessment	UN	G	-Needs internet access to function
23	Vision to vision	Hridaan sharma	4.0.0	UN	2024/06/12	Android 5.0 and up	Physical health	Assessment	UN	G	-
24	Eye patient	Cloud Nine Development	3.1.2	UN	2024/05/08	Android 5.0 and up	-Physical health	-Information, Education -Assessment	UN	G	-Allows sharing -Reminders for medication and appointments - Requires login -Needs internet access to function.
25	Pristine eye hospitals	Pristine eye hospitals	2.0.5	UN	2024	Android 7.0 and up	-Physical health	-Information, Education -Monitoring, Tracking -Advice, Tips, Strategies, Skills Training -Goal Setting	CM	G	-requires login -Allows sharing، - Reminders for eyedrop administration -Needs internet access to function.
26	AR Eye	Yetzer Studio, LLC	2022.1	5	2023	iOS 12.0 or later/ iPadOS 12.0 or later./ App Store	-Physical health	- Advice, Tips, Strategies, Skills training -Assessment -Information, Education	UN	G	-
27	Uveitisoncall	mHealth Wellness LLC	-	5	UN	iOS 15.0 or later, macOS 12.0 or later and a Mac with Apple M1 chip or later, visionOS 1.0 or later	-Physical health	-Assessment - Feedback	UN	17+	-Needs internet access to function.
28	Ocularcheck: acuity exam	Project ORBIS International Inc	2.2.0	3.8	2024	iOS 17.0 or later	-Physical health	-Assessment	UN	G	-
29	Glaucoma simulator	Dares Core d.o.o.	1.0.1	UN	2024	iOS 16.0 or later, iPadOS 16.0 or late, macOS 13.0 or later and a Mac with Apple M1 chip or later	-Physical health	- Feedback	UN	12+	-Needs internet access to function
30	SUPRACOR™ Advisor	Bausch & Lomb Incorporated	-	UN	UN	iOS 11.0 or later, macOS 11.0 or later and a Mac with Apple M1 chip or later, visionOS 1.0 or later	-Physical health	-Assessment	UN	G	-
31	Cataract lens	Chun-Huei Liu	1.1.0	UN	2024	iOS 12.0 or later, iPadOS 12.0 or later, iOS 12.0 or later, macOS 11.0 or later and a Mac with Apple M1 chip or later.	-Physical health	-Assessment - Feedback	UN	12+	-Allows sharing
32	Visionstation	Switt Kongdachalert	-	UN	UN	iOS 13.4 or later, iPadOS 13.4 or later, iOS 13.4 or later, macOS 11.0 or later and a Mac with Apple M1 chip or later, visionOS 1.0 or later	-Physical health	-Assessment	UN	12+	-
33	Vision care- eye exam	Manita Trakansirorut	1.5.2	4.5	2023/02/24	iPhone Requires iOS 15.0 or later. iPod touch Requires iOS 15.0 or later. Apple Vision Requires visionOS 1.0 or later.	-Physical health	-Assessment - Feedback	UN	G	-Allows password-protection - Requires login-Reminder
34	Glaucoma notebook – IOP Note	MONOPOLEAPPS K.K.	1.3	4.6	2018	iOS 15.0 or later, iOS 15.0 or later, visionOS 1.0 or later	-Physical health	-Monitoring, tracking strategies	UN	17+	-Allows sharing، - Reminder
35	Complexcataract	mHealth Wellness LLC	-	5	UN	iOS 13.5 or later, iPadOS 13.5 or later, macOS 11.0 or later and a Mac with Apple M1 chip or later, visionOS 1.0 or later	-Physical health	-Assessment - Feedback	UN	12+	-
36	eyeRx	mHealth Wellness LLC	1.7	5	2021	iOS 13.5 or later, iOS 13.5 or later, macOS 11.0 or later and a Mac with Apple M1 chip or later, visionOS 1.0 or later	-Physical health	-Monitoring, tracking strategies	UN	+ 12	- Reminder
37	Eyedea refractive	Eyedea Educacao e Desenvolvimento em Oftalmologia LTDA	2.4.1	5	2023	iOS 10.0 or later, iPadOS 10.0 or later, macOS 11.0 or later and a Mac with Apple M1 chip or later, visionOS 1.0 or later	-Physical health	-Assessment	UN	17+	-
UN: Unknown NGO: Non-Governmental Organization CM: Commercial G: General											

Based on Table 3, of the 37 reviewed apps, three apps (8%) were developed by mHealth Wellness LLC. These apps had different versions, and four apps (11%) did not report their version. A total of 25 apps (68%) disclosed their ratings; eight apps (22%) received a five-star rating, and 11 apps (30%) received a zero-star rating. Additionally, 16 apps (43%) in 2024 and 17 apps (46%) in earlier years reported their last up date, while four apps (11%) did not.

A total of 25 apps (68%) were designed exclusively for the Android platform, and 12 apps (32%) were available only for iPhone. For the two apps available on both platforms—Eye Patient and Vision Training & Eye Exercise—the Android versions were evaluated as the representative versions to ensure a consistent and non‑duplicative assessment.

Overall, all apps (100%) focused on preventive eye care, targeting physical health. Among these, eight apps, 22% emphasized behavior change by encouraging users to take regular eye breaks. Additionally, two apps (5%) incorporated goal-setting features, as they help users define, track, and manage their personal, behavioral, or health goals, and one app (3%) adopted an entertaining approach by offering engaging tests for children. Also, in 27 apps (73%), an assessment strategy was used to evaluate the users’ visual skills and identify areas for improvement; in 20 apps (54%), information and education were provided to enhance the users’ health and well-being.

A total of 15 apps (40%) offered performance-based feedback during exercises along with advice, tips, strategies, or skills training. Additionally, 13 apps (35%) included monitoring and tracking strategies to follow the users’ progress. Goal setting was implemented in six apps (16%) to improve visual skills. One app (3%) employed a relaxation strategy by suggesting music to promote well-being. Regarding organizational affiliation, one app (3%) was associated with a non-governmental organization (NGO), one app (3%) was commercial, and for 35 apps (94%), the organizational affiliation was unknown. A total of 15 apps (40%) were suitable for all age groups, eight apps (22%) were designed for individuals over 12 years old, and two apps (5%) did not specify a target age group.

Among the reviewed apps, 19 apps (51%) required sending reminders for their functionality, 15 apps (40%) depended on Internet connectivity, 9 apps (24%) allowed sharing features, and four apps (11%) required user login. An intra-class correlation coefficient was calculated to assess inter-rater reliability. The ICC for absolute agreement was 0.98 (95% CI 0.97–0.99), indicating excellent reliability among the raters.

## Discussion

The present study provides a quantitative and qualitative assessment of ophthalmology-related mobile applications across four app stores using the MARS. A total of 37 eligible apps were identified and evaluated, including four Persian-language and 33 English-language apps. Notably, the limited number of Persian-language apps identified in this review—despite the growing demand for accessible digital resources among Persian-speaking populations—may indicate a significant gap in locally tailored tools for eye health education, self-monitoring, and care.

Among the mobile apps focused on eye health, the predominant features encompassed Visual acuity examination, eye examination, preventive eye care, eye exercises, self-examination and patient education, eye health monitoring, guidance on disease risk, explanation of the eye function and simulation of visual impairment, disease management, treatment recommendation, personalized recommendation, recording intraocular pressure progression, calculation of cataract surgery complexity, and refractive surgery calculator.

Reminder functions were among the most common features and may support self-management and improve adherence to recommended routines or treatment regimens [24]. Other frequently observed characteristics included Internet connectivity requirements, sharing options, and account login, which may enhance interactivity but also reduce usability and accessibility for some users [24].

A total of 65 apps were excluded due to technical issues such as failure to open or function properly. Since technical stability is a key dimension of the app quality, recent policy updates also indicate that Google plans to remove low-quality Android apps from Google Play starting September 2024 to improve the overall quality of published applications [25].

By examining the most recent updated apps, only 16 apps (43%) were updated within the past year, whereas 17 apps (46%) had remained un-updated for over one year.

Prolonged lack of updates increases the risk of security vulnerabilities and privacy breaches, underscoring the importance of regular maintenance for safeguarding user data and improving the app performance [26]. Among the 37 reviewed apps, organizational affiliation was not reported for 35 apps (94%), while only one app (3%) was linked to an NGO and one app (3%) to a commercial organization. To address this transparency gap, we recommend that developers of ophthalmology-related mobile apps explicitly disclose their organizational affiliation in the app description, privacy policy, or a dedicated “About” section. Reporting organizational affiliation is an important aspect of transparency and may influence the users’ perceptions of credibility and trust in health-related applications [27]. Regulatory bodies and app marketplaces could further support this by requiring affiliation information as part of submission guidelines or verification processes.

These results align with prior research on eye-care mobile apps, which similarly indicated that very few apps involved collaboration with medical institutions or specialist ophthalmologists in their development, and the majority received infrequent updates—thus limiting timely access to the current eye health knowledge and technological improvements [26].

The overall MARS scores for the evaluated apps ranged from 2.15 (Visionstation) to 4.83 (Eye Patient), with an average score of 3.59, indicating that the overall quality of eye care apps available across the studied app stores is acceptable. Among the four MARS domains, Functionality received the highest mean score (4.24), followed by Aesthetics (3.70), Engagement (3.22), and Information (3.13).

The highest MARS rating was observed in the Functionality subscale, indicating that most apps demonstrated accurate performance and user-friendliness. This finding aligns closely with a recent systematic review on visual acuity apps, where Functionality also received the highest scores [7], suggesting that developers have prioritized this critical dimension to enhance overall user experience and satisfaction [28, 29].

The relatively lower scores in Engagement and Information suggest that many ophthalmology-related mobile applications emphasize basic technical performance and usability, while paying less attention to sustained user motivation and evidence-based educational content. Limited personalization, lack of interactive elements, and insufficient feedback may contribute to weaker Engagement. Similarly, low Information scores may reflect unclear authorship, inadequate scientific referencing, and limited clinical validation or expert involvement in content development. Providing high-quality information can help ensure that users benefit from these applications in a safe and appropriate manner [29]. Similar MARS-based evaluations in other health domains have also reported higher Functionality scores compared with Engagement and Information domains, suggesting that this pattern may reflect a common limitation of many health-related mobile apps internationally [22, 24, 25].

To address these gaps, developers should apply user-centered design strategies to improve Engagement, including personalization, reminders, goal setting, progress tracking, and interactive feedback. Improving the Information domain also requires transparent authorship, use of clinical guidelines and peer-reviewed evidence, clear reporting of content update dates, and involvement of ophthalmologists or academic institutions in content development. Strengthening these factors may enhance the user’s trust, promote sustained use, and improve the overall effectiveness of ophthalmology-related mHealth applications [30].

Scores related to Subjective quality (with an average of 3.13) and Perceived impact (with an average of 3.21) were generally acceptable.

These findings highlight several actionable implications for stakeholders. Developers should prioritize evidence-based content, transparency, and engagement features; clinicians should recommend these apps cautiously due to limited validation; and policymakers should consider minimum standards for privacy, transparency, and clinical evidence reporting.

There was a wide gap in the scores of each subdomain, with Engagement scores ranging from 2.0 to 5.0, Functionality scores from 2.0 to 5.0, Aesthetics scores from 1.33 to 5.0, Information scores from 1.85 to 4.85, Subjective quality scores from 1.25 to 5.0, and Perceived impact scores from 1.0 to 4.66. This variation indicates that some apps have high quality, such as “Eye Patient” with an average score of 4.83, while others have low quality, such as “Visionstation” with an average score of 2.15.

This significant disparity aligns with the findings from prior systematic reviews of visual acuity and eye-care apps, which similarly reported wide ranges in subdomain scores (e.g., Functionality 2.00–5.00, Subjective Quality 1.00–5.00) and emphasized the need for greater attention to improve the mHealth apps’ features, evidence base, and overall effectiveness for health promotion and clinical utility [7, 26]. This significant difference in quality shows that the existing mHealth apps on the markets require greater attention and improvement in order to offer more advanced and effective features and capabilities for health promotion [28].

To the best of our knowledge, the majority of the 37 ophthalmology-related mobile apps evaluated in this review lack robust scientific validation or published evidence supporting their clinical effectiveness, diagnostic accuracy, or practical utility in eye care.

Reference [23] provides preliminary evidence of the effectiveness and usability of the " WHOeyes” app in visual acuity screening among the student population and can serve as preliminary scientific support for the use of this app in population-based studies. This appn aligns well with the Functionality domains of the MARS, given its validated accuracy. It primarily serves as a straightforward screening tool rather than an engaging or gamified mHealth app, and thus its Engagement features appear limited.

In a study [31], the “Eye Exam” app is also mentioned; however, it only introduces and lists these programs without providing scientific evidence of their effectiveness. Thus, the majority remain not validated, limiting their safe adoption for self-monitoring, teleophthalmology, or population-based screening. These observations align with broader trends in mHealth systematic reviews, which consistently highlight insufficient clinical validation and professional oversight in most eye-care apps, calling for more rigorous testing and transparency to ensure evidence-based use by clinicians and patients [1, 7, 26, 32].

### Limitations and future work

One of the main limitations of this study is that mobile apps are dynamic and frequently updated. Consequently, the evaluations presented here represent a snapshot in time and may not reflect the features, functionality, or performance of the current app versions. Additionally, the lack of transparency in the app store search algorithms limits the reproducibility of the search, as results can vary significantly depending on the device, geographic location, language settings, and user personalization [15, 22]. Although strict inclusion and exclusion criteria were used, only 37 applications met the eligibility criteria. Consequently, the included apps may not represent the entire range of mobile apps available for ophthalmology, limiting the generalizability of the results. Additionally, the search was limited to the app stores, which may have excluded relevant apps available through other sources such as web-based tools, introducing potential sample bias. Due to the growing popularity of Web-based apps, we believe it is crucial to include these apps in future research projects [33]. In spite of the fact that we attempted to broaden the search scope as much as possible by using a comprehensive set of conceptual and domain‑relevant keywords, the inherent limitations of app distribution platforms—such as complex ranking algorithms, developers’ heterogeneous app store optimization (ASO) strategies, and personalized search results—prevent complete assurance that all relevant applications can be retrieved.

Some applications were not included in this review because they were accessible only to users in specific countries and required registration with a national identifier or other country-specific credentials to access their features. Among the limitations of this study is that the app quality was evaluated without incorporating feedback from end-users. While the MARS provides a strong expert-based framework for assessment, it may not fully reflect the users’ subjective experiences or perceptions. Including user feedback in future investigations—through instruments such as the user version of MARS (uMARS) or direct patient surveys—could yield a more comprehensive understanding of the effectiveness and usability of the app.

## Conclusion

While ophthalmology apps hold promise, current scientific evidence and quality assessments do not support broad claims of high quality across all domains. Future development must focus on rigorous clinical validation, improved information quality, and standardization to ensure these tools are both safe and effective for eye health promotion, self-monitoring, and clinical use. Based on the MARS evaluation, ophthalmology-related mobile applications generally demonstrated good performance in the Functionality domain, and the performance in the Information, Engagement, and Aesthetics domains was acceptable. Specifically, the majority of apps lacked evidence-based content and valid scientific support, and only one app (“WHOeyes”) showed preliminary evidence of effectiveness in a peer-reviewed study. While certain apps may be beneficial for limited purposes (e.g., visual acuity screening or eye exercises), the inconsistent overall quality and notably low Information domain scores undermine the reliability and clinical safety of most currently available ophthalmology apps. Therefore, the following recommendations are proposed:

- Clinicians should only recommend apps that have demonstrated acceptable quality across multiple MARS domains—especially Information and Functionality—and preferably those with published clinical validation.
- Developers must prioritize evidence-based content aligned with current ophthalmology guidelines, improve user engagement through interactive and adaptive features, and provide transparent validation data. Localization (Persian/English) and user-centered design are also essential.
- Policymakers should establish regulatory frameworks and certification systems (e.g., based on MARS or similar standards) to assess and approve ophthalmology apps before public release. Funding and interdisciplinary collaboration should be directed toward clinically safe, well-validated digital tools.

## Supplementary Information

Below is the link to the electronic supplementary material.

Supplementary Material 1

Supplementary Material 2

Supplementary Material 3

Supplementary Material 4

## Data Availability

All data used in this study are included in the text of the article.

## Abbreviations

MARS: Mobile App Rating Scale
mHealth: mobile Health
WHO: World Health Organization
iOS: iPhone Operating System
NGO: Non-Governmental Organization
PRISMA: Preferred Reporting Items for Systematic Reviews and Meta-Analyses
PROSPERO: Prospective Register of Systematic Reviews
ICC: Intraclass correlation
ASO: App store optimization
