# Supplementary material for: Mobile applications for ophthalmology: a quantitative and qualitative analysis
Source: BMC Health Serv Res. 2026 Jul 23;26:1433. doi: 10.1186/s12913-026-15199-2 (PMC13681809; doi:10.1186/s12913-026-15199-2)
Supplement: Supplementary file 2 [file 12913_2026_15199_MOESM2_ESM.docx]

Bazaar store accessed on July31th to August 15th, 2024 (1464)

ocular (17)

vision (262)

Ophthalmology (4)

Eye (364)

چشم (405)

چشم پزشکی (412)

Remove duplicates (251) Apps that could not be installed (24)

Non-repetitive apps (1189)

Checking apps based on title and description and removing apps(1067) Apps that are not related to ophthalmology (662) Apps that are in the form of games (405)

Apps assessed for eligibility(122)

. Language other than English or Persian (0 ) . Non-free apps (21) . Apps that are solely related to adjusting the brightness of the phone(36) . App require in-app purchases (31) . App not be publicly accessible (2) . Non-interactive apps that do not engage users effectively (15) . Apps related to selling glasses (0)

Installing and checking the content of the apps(17)

**Supplementary2. The selection process of mobile apps from the Cafe Bazaar store**
